# Human Herpesvirus-8 Negative Multicentric Castleman Disease in a Patient with Human Immunodeficiency Virus Treated with Highly Active Antiretroviral Therapy and Chemotherapy

**DOI:** 10.7759/cureus.5530

**Published:** 2019-08-30

**Authors:** S. Caleb Freeman, Janani Baskaran, Maryam Gbadamosi-Akindele

**Affiliations:** 1 Dermatology, Creighton University School of Medicine, Omaha, USA; 2 Internal Medicine, Creighton University School of Medicine, Omaha, USA

**Keywords:** hiv/aids, multicentric castleman's disease, highly active antiretroviral therapy (haart), hhv-8

## Abstract

Multicentric Castleman disease (MCD) is a rare lymphoproliferative disorder with a high mortality rate in undiagnosed patients. Traditionally, human immunodeficiency virus (HIV) positive MCD occurs due to infection with human herpes virus-8 (HHV), which is thought to play a role in the pathogenesis of MCD. We present the case of a 49-year-old woman who was referred to our oncology clinic for generalized lymphadenopathy in a waxing and waning pattern for the last four years. She was found to be HIV positive. Here we report a rare case of HIV-positive, HHV-negative MCD that responded to prompt treatment with highly active antiretroviral therapy (HAART) followed by chemotherapy as evidenced by improved CD4+ T cell numbers and reduction in lymphadenopathy. The findings in this HHV seronegative patient may indicate an alteration in the virulence and tropism between HHV and HIV, and further demonstrate the need for continued investigation into the pathogenesis of Castleman disease.

## Introduction

Multicentric Castleman disease (MCD) is a rare lymphoproliferative disorder that commonly presents with lymphadenopathy and traditional "B" symptoms such as fever, night sweats, and weight loss. The pathophysiology of MCD has overlapping features of both autoimmune and infectious processes. Furthermore, MCD can be broken into two predominant subtypes: 1) human herpesvirus-8 (HHV) positive MCD and 2) HHV negative or idiopathic MCD [1]. Approximately 50% of HHV MCD cases are also associated with HIV, which changes the treatment course considerably [2]. HIV-positive MCD typically occurs in conjunction with HHV infection, which is thought to play a role in the pathogenesis of MCD [3-4]. After an extensive literature search, we believe we are reporting the first case of HIV-positive, HHV-negative MCD.

## Case presentation

A 49-year-old female was referred to our oncology clinic for generalized lymphadenopathy in a waxing and waning pattern for the last four years. A complete blood count revealed leukopenia with a leukocyte count of 2.5 k/µL (normal range: 4-11 k/µL). CT scan revealed bilateral axillary, supraclavicular, subpectoral, submental, retroperitoneal, and periaortic lymphadenopathy (imaging was performed at an outside facility and thus original images were not included in this report). An excisional right axillary lymph node biopsy was performed. Histology showed an atypical lymphoid infiltrate with an immunoglobulin λ (Igλ) gene rearrangement suggestive of Castleman disease. Bone marrow biopsy showed no abnormalities. HIV viral load was 104 log copies/mL (normal range: not detected) and absolute CD4+ T cell count was 84 cells/µL (normal range: 500-1400 cells/µL). Of note, immunostain for HHV was negative. 

Multicentric Castleman disease has a nonspecific presentation that includes waxing and waning lymphadenopathy, night sweats, fatigue, and fevers. As a result, malignant lymphoma was high on the differential. In addition, autoimmune disease-associated lymphadenopathy and HIV-associated lymphadenopathy were also considered. These diagnoses have a similar presentation. Therefore, further workup was indicated. Ultimately the final diagnosis was confirmed by biopsy which showed characteristic atypical lymphoid infiltrate with Igλ rearrangement. Consequently, we were able to differentiate between MCD and other causes of generalized lymphadenopathy. 

The patient was first started on antiretroviral therapy, consisting of elvitegravir, cobicistat, emtricitabine, and tenofovir. Given her immunocompromised state, chemotherapy for MCD began after HIV titers fell and CD4+ T cell count increased. Chemotherapy treatment consisted of rituximab and etoposide, once weekly for four weeks. Highly active antiretroviral therapy (HAART) quickly restored our patient’s CD4+ T cell numbers and decreased viral load, as demonstrated in Table 1. 

**Table 1:** Timeline of patient treatment. Patient’s CD4+ T cell count and HIV viral load as measured during therapy for HIV and MCD.  *designates beginning HAART therapy.  **designates initiation of rituximab.

	Month 1	Month 3	Month 7	Month 12	Month 13
Absolute CD4+ T cell count (cells/µL)	84*	116**	201	241	359
HIV viral load (log copies/mL)		104	<40	<40	Not detected

There was a clinical reduction in lymph node size 10 days after treatment with chemotherapy was initiated. Furthermore, eight months after the completion of chemotherapy the patient was determined to be in remission. Remarkably, four years later the patient shows no signs of relapse.

## Discussion

Because of the limited number of cases of MCD, the incidence is hard to quantify. However, a recent estimate suggests that 4-7 cases of MCD occur per million people each year [5]. The diagnosis of MCD can be also difficult due to the nonspecific findings in the history and physical exam in patients with MCD. Common presenting symptoms include fever, night sweats, weight loss, and widespread lymphadenopathy [6]. The nonspecific nature of presentation is further complicated by the manner in which it closely mimics lymphoma on positron emission tomography (PET) scan and CT [7-8]. Diagnosis, therefore, must be confirmed with a biopsy. Histologically, MCD demonstrates large, abnormal plasmablasts within the mantle zone [9]. In addition, these plasmablasts show λ light-chain restriction [10]. Following a biopsy, immunostaining is typically performed for HHV, which was notably negative in our patient despite her positive HIV status. Our patient’s case exhibits several extraordinary features, primarily for being the first reported case of HIV-associated Castleman disease in an HHV-seronegative patient. Although five-year overall survival for MCD is over 50%, this patient's state of complete remission is rare considering that the three-year disease-free survival rate in idiopathic MCD is 46% and the three-year disease-free survival for HIV+ patients with MCD is just 28% [11-12]. 

Treatment for MCD traditionally included chemotherapeutic agents such as etoposide or vinblastine with limited success [13]. Recently, modalities have improved to include the anti-CD20 humanized antibody rituximab and biologic agents such as the anti-IL-6 chimeric antibody siltuximab [14]. Our patient’s excellent response to therapy and remarkable remission is possibly related to her HIV status. This result was likely influenced by the timely diagnosis and institution of HAART followed by chemotherapy and continued monitoring. Institution of HAART in HIV positive MCD patients was initially reported to cause deterioration of MCD, but most case reports now demonstrate improvement in lymphadenopathy with HAART even prior to the initiation of chemotherapy [13]. This may be attributable to newer anti-retroviral therapies.

## Conclusions

The findings in this HHV seronegative patient may indicate an alteration in the virulence and tropism between HHV and HIV, and further demonstrate the need for continued investigation into the pathogenesis of Castleman disease. This patient’s excellent clinical response also demonstrates the importance of a team-based approach to patient care, involving primary care physicians, oncologists, pathologists, and infectious disease specialists.
